# La luxation erecta bilatérale: à propos d'un cas

**DOI:** 10.11604/pamj.2015.22.316.7456

**Published:** 2015-12-02

**Authors:** Tarik Madani, Redouane Hani, Mohamed Amine Karabila, Mohammed Kharmaz, Mohamed El Ouadghiri, Abdou Lahlou, Mly Omar Lamrani, Ahmed El Bardouni, Mustapha Mahfoud, Mohamed Saleh Berrada, Mouradh El Yaacoubi

**Affiliations:** 1 Service de Traumatologie-Orthopédie au CHU IBN-Sina, Rabat, Maroc

**Keywords:** Luxation bilatérale, erecta, épaule, bilateral dislocation, erecta, shoulder

## Abstract

La luxation erecta est une lésion rare, elle est encore plus rare quand elle est bilatérale. Nous rapportons un cas de luxation erecta bilatérale chez un sportif victime d'une chute lors d'une séance de gymnastique. Le patient s'est présenté aux urgences avec une attitude d'abduction irréductible des deux épaules, l'examen vasculo-nerveux était normal. La radiographie a confirmé le diagnostic d'une luxation erecta bilatérale associée à une fracture des deux tubercules majeurs. Le patient a bénéficié d'une réduction orthopédique et d'un bandage coude au corps. Le suivi a montré un score de l'UCLA à 30 points. L'objectif de notre travail est d'insister sur la rareté de la luxation erecta bilatérale et de rappeler sa particularité clinique, thérapeutique et évolutive.

## Introduction

La luxation de l’épaule, est définie par une perte de contact totale et permanente de la tête humérale avec la cavité glénoïde de la scapula se produisant au décours d'un traumatisme. C'est une des urgences thérapeutique à cause du risque vital du membre (compression d’éléments vasculo-nerveux) et aussi fonctionnel (déformation articulaire, instabilité, arthrose). L'attitude vicieuse diffère selon la forme anatomopathologique, le membre en abduction irréductible est pathognomonique de la luxation erecta. Nous rapportons le cas d'une luxation erecta bilatérale chez un jeune patient survenue lors d'une chute les deux bras en abduction. L'objectif de notre travail est d'insister sur la rareté de la luxation erecta bilatérale et de rappeler sa particularité clinique, thérapeutique et évolutive.

## Patient et observation

Nous rapportons le cas d'un patient de 19 ans qui a été admis aux urgences du CHU de Rabat pour douleur et impotence fonctionnelle totale des deux épaules survenue lors d'une séance de sport (gymnastique) après une chute sur les barres parallèles les deux bras en abduction le patient n'avait pas d'antécédent d’épaule instable.

**Figure 1:**
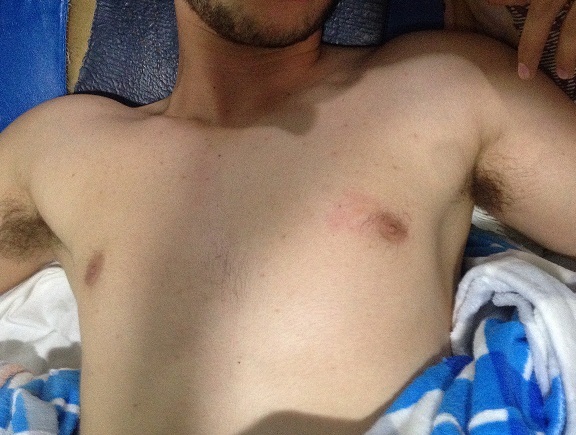
Attitude irréductible des deux épaules en abduction

**Figure 2:**
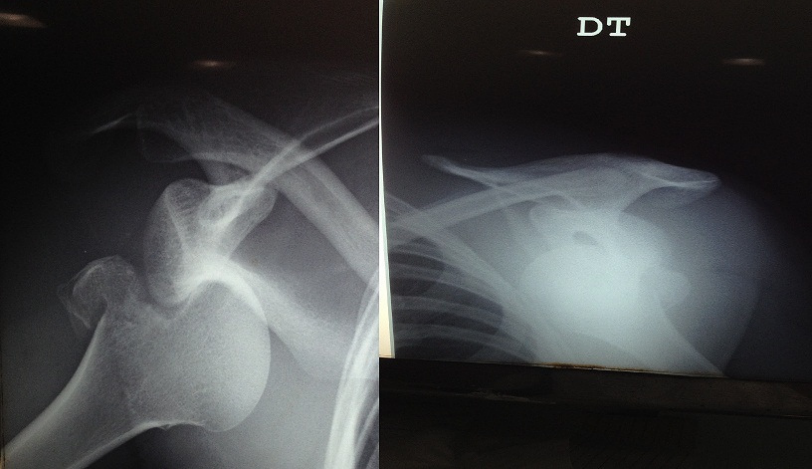
Position sous-glénoïdienne de la tête humérale associée à une fracture des deux tubercules majeurs

L'examen clinique trouve une déformation des moignons des deux épaules avec les deux membres supérieures en abduction et une impossibilité de ramener le coude au corps (Figure 1). L'examen vasculo-nerveux est normal. La radiographie standard des deux épaules a montré une luxation erecta bilatérale avec une position sous-glénoïdienne de la tête humérale associée à une fracture des deux tubercules majeurs (Figure 2).

**Figure 3:**
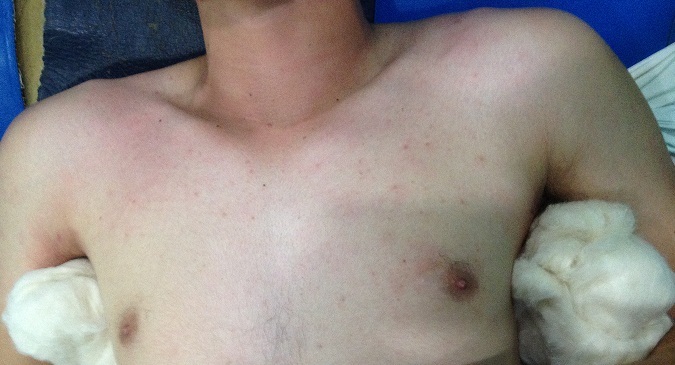
Image montrant la réduction de la luxation bilatérale

Le patient a bénéficié d'une réduction sous sédation avec succès (Figure 3), elle consistait en une traction dans l'axe du membre avec un bondage coude au corps pendant 3 semaines puis adressé au service de kinésithérapie pour rééducation. Le contrôle après six mois a objectivé une élévation antérieure de 150°, en se basant sur l’échelle de notation de l'UCLA [1] le patient avait un score de 30 points.

## Discussion

La luxation erecta est une entité relativement rare, elle représente seulement 0,5% de toutes les luxations de l’épaule [2, 3], la luxation erecta bilatérale est encore plus rare, seulement quelques cas ont été publiés jusqu’à aujourd'hui [4–6]. La présentation clinique était pathognomonique chez notre patient avec le mécanisme chute sur les bras en abduction, la fixation irréductible des épaule en abduction et la tête humérale palpable sous la glène contre la cage thoracique [4, 5], Gagey et al. ont décrit ce mécanisme, à propos de 32 luxations erecta expérimentales [7], par une simple élévation rotation externe du membre. Le terrain d'hyperlaxité ligamentaire a été rapporté dans plusieurs séries [8, 9]. Les accidents de la voie publique représentent l’étiologie principale suivi des accidents de sports [10, 11].

La réduction de la luxation par la technique de traction-contre traction réalisée avec succès pour les deux épaule montre l'efficacité de la technique elle consiste en une traction du bras dans l'axe du membre pendant que l'aide applique un contre appui sur le thorax. Le bras ensuite ramené en adduction et une immobilisation coude au corps est gardé pendant 3 semaine. Une radiographie post-réduction doit être faite afin de confirme la réussite de la réduction et de décelé d’éventuelle fracture iatrogène.

Nous n'avons pas noté de complications vasculo-nerveuse chez notre patient, pourtant des cas de lésions de l'artère axillaire et du plexus brachial ont été observés à cause de la proximité de l'articulation gléno-humérale de ces deux éléments nobles, dans la série de Mallon et al. [6] comportant 86 observations, ont été rapportées une atteinte du nerf axillaire dans 60% des cas et une atteinte de l'artère axillaire dans 3% des cas. Garcia et al. ont rapporté un cas de luxation erecta bilatérale compliqué d'une thrombose de l'artère axillaire imposant un traitement anticoagulant [12].

Le pronostic de la luxation erecta bilatérale était bon chez notre patient vu le score UCLA obtenu au sixième mois malgré les lésions osseuses associées, mais pour d'autres auteurs [13] le pronostic était moins bon.

## Conclusion

La luxation erecta est une lésion rare. Il faut y penser devant toute luxation de l’épaule en hyper abduction car elle peut être prise pour une luxation antérieure. Les lésions associées sont fréquentes, notamment les fractures du tubercule majeur. Le pronostic est le plus souvent favorable. La réduction orthopédique est la technique de choix et la rééducation doit être précoce.
